# Observation of monochromatic and coherent luminescence from nanocavities of GaN nanowall network

**DOI:** 10.1038/s41598-021-88660-3

**Published:** 2021-04-30

**Authors:** Danish Shamoon, Kishor Upadhyaya, Sonnada M. Shivaprasad

**Affiliations:** 1 International Centre for Materials Science (ICMS), Jawaharlal Nehru Centre for Advanced Scientific Research (JNCASR), Bangalore, 560064 India; 2 Centre for Materials Science, K. L. E. Technological University, Hubballi, Karnataka 580031 India

**Keywords:** Materials science, Nanoscience and technology, Optics and photonics

## Abstract

Scaling-down the size of semiconductor cavity lasers and engineering their electromagnetic environment in the Purcell regime can bring about spectacular advance in nanodevices fabrication. We report here an unprecedented observation of a coherent Cathodoluminescence from GaN nanocavities (20–100 nm). Incident lower energy (< 15 kV) electron beams excite the band edge UV emission from the walls of the network whereas for higher energies, the emitted photons are spontaneously down converted into NIR and preferentially emerge from the nanocavities. Non-centrosymmetric structure of GaN and its nanowall geometry together facilitate this unique observation which is substantiated by our numerical results. At cryogenic temperatures, an intense and narrow laser-like NIR beam emanates out of the nanocavities. The work promises the possibility of fabrication of very high density (over 10^8^/cm^2^) cavity lasers that are addressable by simple deflection and tuning of incident electron beams.

## Introduction

Since their inception in 1960s, compact photonic components with semiconducting coherent light sources (especially semiconductor lasers) have revolutionized the fields of optical communication, data storage and processing, ranging (LiDAR), printing, spectroscopy, bio-imaging and sensing. Since then, developing laser sources with high speed, intensity and power gain has been the subject of the intense research across the globe. One strand of such research focuses on reduction of physical dimensions of lasers which would result in a scalable nanolaser with increased cavity-emitter interaction (Purcell effect), high integration density and speed, low threshold current and low input power. This would require fabrication of small lasers which can confine the carriers and photons within the cavity without additional loss of photons or carriers thereby reducing the cavity round trip time as well as the device capacitance. Different nanolasers with microdisks, photonic crystals, plasmonics, nanowires, metallic and metal-dielectric resonators have been proposed recently^1^. But progress towards realizing the ultimate nanolaser is challenged by scaling down the size of the laser cavity below that of diffraction limit and nullifying the ohmic losses at optical frequencies. One of the best ways of overcoming these challenges is to fabricate laser diodes containing vertical-cavities by vertically distributing semiconductor Bragg reflectors to emit light along the direction normal to the surface of the semiconductor wafer. These Vertical-Cavity Surface Emitting Lasers (VCSELs) have recently attracted a lot of interest and hold edge over other type of lasers because of their high modulation speed at low threshold currents and high quantum efficiency which is primarily due to the small size of the vertical cavities^2–5^. VCSELs can generate circular and non-astigmatic beam because of their symmetrically transverse device structure which is most suitable for feeding light to optical fibers and other optical components. Surface emission enables fabrication of large scale two dimensional arrays of vertical cavities at wafer level which lowers the manufacturing cost with better optical gain. GaAs and its alloys have been conventionally used for fabricating VCSEL based laser diodes but suffer from low threshold current and high refractive index and cannot generate wide spectrum of wavelengths (especially in UV range) which limit the device performance. Hence, search for a semiconductor material with small sized vertical cavities distributed uniformly in two dimensions over a large wafer surface to produce the ultimate nanolasers is still on.

Recently, GaN and its alloys have been preferably used over other semiconductors to fabricate such VCSELs because of their wide band gap, chemical resistance, high temperature/high power capability and high electron saturation velocity. Theoretical and experimental studies on III-nitride based waveguides have shown their suitability in the applications of non-linear optics like second harmonic generation (SHG) and parametric down conversion due to their active integration capacity and low material dispersion^6,7^. But tunable frequency emission from the same nanostructured thin film and spatially-resolved nanoscale nonlinear response which can enhance the bandwidth as well as the intensity of continuous wave generation and which is also crucial for improving the performance of VCSEL devices has not been explored so far.

In previous reports^8,9^, it has been shown that by kinetically controlling the growth parameters for the construction of semiconductor thin films using Molecular Beam Epitaxy (MBE), we can form a very interesting single-crystalline morphology consisting of polygonal shaped cavities bound by steep-wedge shaped nanowalls by a misfit relaxation pathway forming open screw dislocations. This porous structure called as a Nanowall Network (NwN) is single crystalline in nature and yields a highly intense band edge emission, with no defect related peaks. Cross-sectional images (not shown) of these films show that these nanowalls are ≈ 1 µm in height, ≈ 5 nm wide at the apex and 100–150 nm wide at the bottom, forming polygonal shaped cavities in the valleys. The lateral opening width of these cavities can vary from 20 to 200 nm at the top and less than 10 nm at the bottom of the valleys.

In this work we provide experimental evidence of intense photon emission emanating from the nanocavities of the NwN morphology of w-GaN upon a highly localized excitation by a high energy electron beam. Spectral investigation reveals that the emission comprises not only the known ultraviolet (UV) component due to the band gap of 3.41 eV but also a near-infra red (NIR) component that seems to interact with UV component and get influenced by the applied beam energy. We also present numerical modelling results that support the creation of whispering gallery modes (WGMs) and non-linear frequency conversion. We argue that the characteristic NwN morphology plays an important role in sustaining WGMs and also providing an escape route through the nanocavities preferentially for the NIR emission.

## Results and discussion

### Intense emission from nanocavities

**Figure 1:**
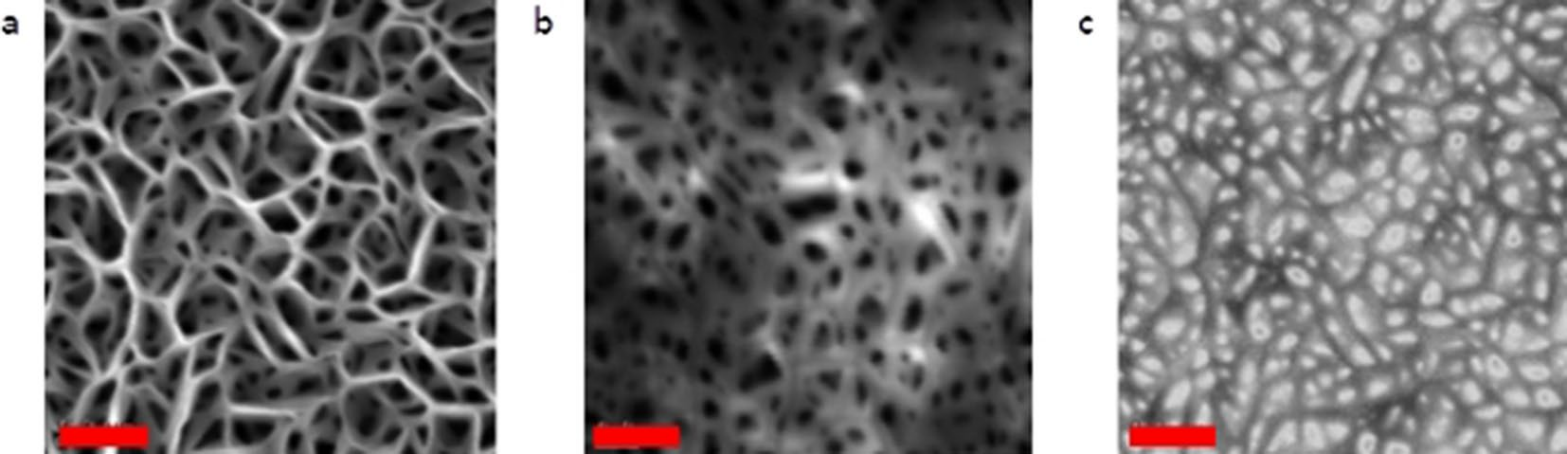
Spatial segregation of emission from nanowalls and nanocavities. (**a**) FESEM plan-view image of w-GaN NwN, (**b**) Panchromatic CL map obtained at room temperature 300 K with applied beam energy of 10 kV and (**c**) 25 kV. Scale bar corresponds to 400 nm.

Photon emission properties of semiconductor thin films are often studied with Photoluminescence (PL), Electroluminescence (EL) and Cathodoluminescence (CL). CL is distinguished from the other methods as it offers a much higher spatial resolution, owing to the use of a high energy focused electron beam as the source for excitation of the material. A beam spot of approximately 1–5 nm locally excites the sample surface generating electron–hole pairs in the direct band-gap semiconductor. Upon recombination of these pairs, photons are emitted consequently which can be recorded. The excitation depth inside the material is controlled by the applied electron beam energy. A CL map offers a direct visual observation of regions on the sample surface from which photons are emitted with a lateral resolution close to the beam spot size (few nm). Field emission scanning electron micrograph (FESEM) image of the sample surface of w-GaN NwN is shown in Fig. 1a. CL maps obtained at an applied beam energy of 10 kV and 25 kV reveal a remarkable difference in the spatial contrast of panchromatic emission intensity detected in the range of 250 to 900 nm. A mere visual comparison of the SEM image with each of the two CL maps shown here intriguingly shows that the photons emanate from the walls in case of lower beam energy (10 kV) as seen in Fig. 1b, whereas in case of higher beam energy (25 kV), the emission is from the nanocavities, as evident from Fig. 1c. This unique emission behavior is further investigated next.

### Non-linear frequency conversion and lasing action

**Figure 2:**
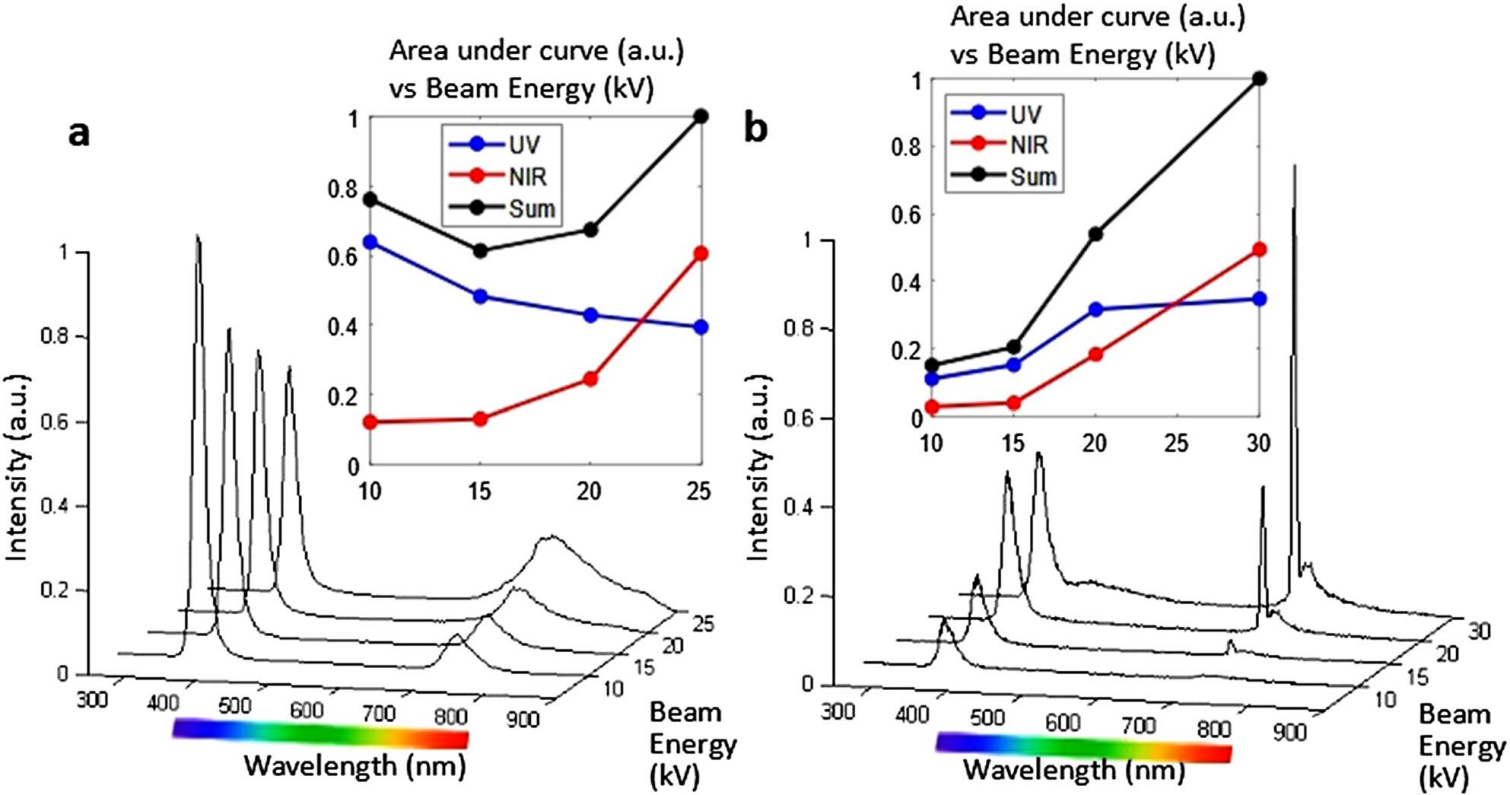
CL spectra for w-GaN NwN vs applied electron beam energy at (**a**) room Temperature (300 K) and (**b**) cryogenic temperature (≈ 93 K). Insets show the area under the curve of UV and NIR components.

While panchromatic CL mapping provides a visual evidence of the ‘hot-spots’ of photon emission. The spectral content of the CL reveals another interesting aspect that the photon emission from the w-GaN NwN is not solely due to its expected band-edge emission. Figure 2a shows the CL spectra obtained at room temperature 300 K. Apart from its narrow UV component peaked at 364 nm, the spectra also contains a sizeable and broad NIR component peaked around 727 nm. Interestingly, the UV and NIR components seem to be non-linearly coupled as suggested by the decrease in UV intensity and increase in NIR intensity as the beam energy is varied from 10 to 25 kV. The area under the curve for each component, i.e. UV and NIR, also corroborates this point as shown in the inset of the same figure. In particular, at 25 kV, the area under the NIR curve surpasses that of the UV curve. Thus, it clearly indicates that the intense emission from the nanocavities is richer in NIR than UV, even though both are present. Additionally, monochromatic CL maps that are detected only for particular frequency such as 363 nm or 725 nm (shown in Supplementary Fig. S1) also corroborate this point (with a drawback of poor image contrast for monochromatic CL maps) that the UV component is richer from the walls while the NIR component is richer in the nanocavities. Since GaN NwN is defect free and single crystalline^8,9^, the role of defects and impurities is ruled out. So, the NIR emission can be attributed to the non-centrosymmetric structure of w-GaN that supports non-linear second order polarization and associated optical processes. This point is theoretically examined in the next section where the role of morphology is examined theoretically. Emission due to defects is commonly observed in the flat film epilayer of w-GaN (i.e. the thin film which is not nanostructured). For comparison, CL data for an epilayer is shown in Supplementary Fig. S2 and it shows a strong band edge emission at 364 nm, a defect band around 580 nm and 440 nm, and a very small NIR emission peaked at 730 nm. In case of epilayer, there was no change in the features of NIR emission spectra with increasing incident electron energies which suggests that the morphology of NwN might be playing a role in the coupled interaction of the UV and NIR components.

Since the phonon effects are reduced at low temperatures and enhance the excitonic emission efficiency, the CL spectra and spatially resolved maps of the NwN sample that is held at low temperature (≈ 93 K) are also obtained. In this case we observe that the UV emission is peaked at 355 nm while a NIR emission maximum was at 707 nm. As evident from Fig. 2b, the relative intensity of both the emission bands again observed changes (inter-conversion of UV into NIR) with varying electron beam energy. Low temperature CL measurements interestingly show that the intensity of the band edge emission (UV emission) peaked at 355 nm also increases steadily as the incident beam energy is increased up to 20 kV and saturates for the higher energies studied. However, the NIR band continuously increases with increasing electron energy and at 15 kV a new and sharp emission peak emerges at 692 nm and rapidly increases in intensity as the incident beam energy increases. The FWHM of the 692 nm peak becomes 5 nm at 30 kV. This sharp and narrow NIR emission peak is very interesting that suggests a laser-like population inversion and its origin needs to be explored further in future. At present, we use numerical modelling along with plausible assumptions to partially examine these observations in the next section. However, it is worthwhile to note that nonlinear optical interactions have been previously exploited and optimized in case of single hexagonal GaN micropyramid to realize microphotonic white light sources & coherent tunable UV–VIS sources using spatially controlled excitation without any change in the material properties^10^. The spatially controlled excitation in that work is however different from our case. It is rather due to the confinement of the enhanced mode at the tip of the geometry. Second harmonic generation (SHG) and multiphoton luminescence were reported in that work in which UV and Yellow emission is observed for excitation above and below half band gap energy.

### Role of morphology in emission from cavity and lasing action

To understand the frequency conversion and spatial segregation of the emission from w-GaN NwN, we note that w-GaN has a non-centrosymmetric structure that supports second order nonlinear optical processes^6,7^. Re-stating from our results that even though the epilayer has shown the presence of NIR component, it did not show any interaction with the UV component while the beam energy was varied. So, it suggests that the NwN morphology has something interesting to do with facilitating the non-linear interaction with UV component and the spatial segregation of the emission making the walls richer in UV emission and the nanocavities richer in NIR emission. A rigorous theoretical analysis of Spontaneous Parameteric Downconversion (SPDC) in simpler 1D GaN/AlN layered media can be found elsewhere^11^. Here, we focus on simpler modelling in two parts for examining the role of morphology. In order to model the non-linear frequency conversion, we invoke the second-order susceptibility in material polarization term that supports three-wave mixing and consider interaction among three frequencies ω_1_, ω_2_ and ω_3_ where ω_3_ is UV and ω_1_ and ω_2_ are the NIR frequencies. The wave equation obtained from Maxwell’s equation incorporating the nonlinear polarization up to the second order is shown below.

1 $${\nabla }^{2}E-\nabla \left(\nabla .E\right)-\mu \varepsilon \frac{{\partial }^{2}E}{{\partial t}^{2}}=\mu \frac{{\partial }^{2}{P}_{NL}}{{\partial t}^{2}}$$

**Figure 3:**
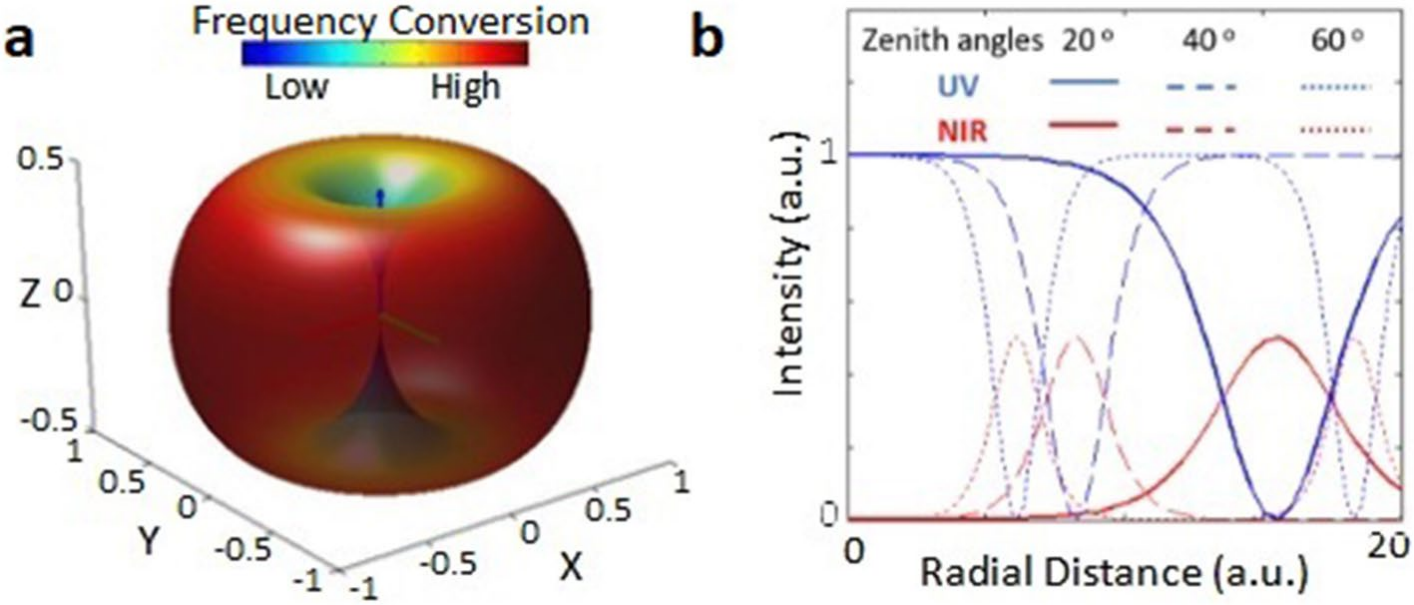
Non-linear interaction of waves leading to interconversion of UV and NIR from a single point of emission in the infinite material (**a**) Angular part of the strength of non-linear frequency conversion for w-GaN, (**b**) Numerical solutions showing intensity (of UV and NIR) vs radial distance travelled by a wave from the point of emission within the material at three specific zenith angles.

Further details are presented in the Supplementary text. We consider a single point of emission. We also assume that the pump wave is generated within the nonlinear material from band-edge CL emission and undergoes self-frequency conversion and may generate emission patterns of a phased-array of atomic dipoles. Self-frequency conversion has been previously reported in other materials as well^12,13^. Analytical derivations yield a set of three frequency-coupled wave equations that are derived assuming specific energy conservation, ω_3_ = ω_1_ + ω_2_. Though, this approach is traditionally used for sum frequency generation, it can also be applied for parametric down conversion^14,15^. Using the d-tensor for *6 mm* class of hexagonal crystal symmetry, the conditions for type 1 phase matching are employed. The final set of simplified equations that are numerically solved is as follows:

2a $$\frac{{\partial E\left( {\omega_{1} } \right)}}{\partial s} = m*E\left( {\omega_{3} } \right)E^{*} \left( {\omega_{2} } \right),$$

2b $$\frac{{\partial E\left( {\omega_{2} } \right)}}{\partial s} = m*E\left( {\omega_{3} } \right)E^{*} \left( {\omega_{1} } \right)$$

and

2c $$\frac{{\partial E\left( {\omega_{3} } \right)}}{\partial s} = m*2E\left( {\omega_{1} } \right)E\left( {\omega_{2} } \right),$$

where *E* denotes the amplitude of the frequency, *s* is the distance travelled by the pump wave (band-edge emission) inside the material along a given direction from the point of emission and *m* denotes the strength of frequency conversion which is equal to ${d}_{31}sin\theta$ for type 1 process. θ is the zenith angle between the z-axis and direction of propagation of pump wave. The strength of frequency conversion is dependent on the zenith angle and different classes of crystal structure symmetries. Figure 3a shows the angular part of the strength. This essentially means that if the wave corresponding to the band-edge emission point travels along the vertical axis there is no frequency conversion at all but if it travels at any angle up to 90 degrees from the vertical zenith then the frequency conversion happens being strongest along the transverse axes. Accordingly, the intensity vs radial distance travelled by both UV and NIR emissions at zenith angles of 20, 40 and 60 degrees is depicted in Fig. 3b. In other words, the pump wave (UV) that propagates along the z-axis will not undergo any conversion. If it propagates closer to z-axis, it will need to travel a larger distance to get converted into NIR whereas if it propagates close to the transverse (xy) plane, it will get converted into NIR in much shorter radial distances. All the frequency conversion happens within the material which means that NwN morphology can influence this frequency conversion.

**Figure 4:**
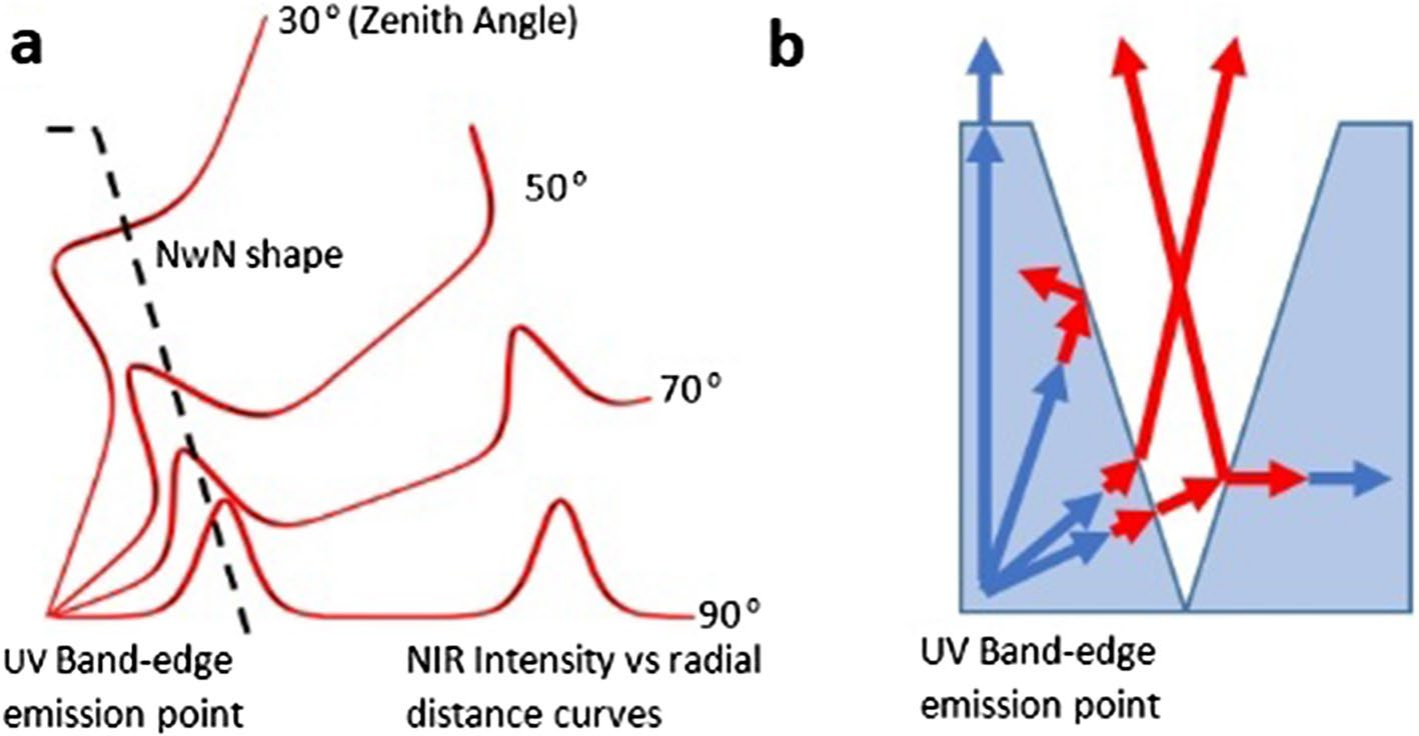
Role of morphology in spatial segregation of UV and NIR from the walls and nanocavities respectively (**a**) Illustration depicting the NIR emission intensity curves as shown in Fig. 3 (b) inside an infinite material from the point of band-edge emission rotated at their respective specific zenith angles, the dashed line indicates a half nano-wall (side view) so that the frequency conversion stops at the boundary if the wave escapes out or if it gets reflected back inside then frequency conversion continues in a new direction, (**b**) Schematic showing inter-conversion of UV-NIR within a nanowall and how UV emanates from the top of the walls while NIR emanates from the bottom of the nanocavity.

NIR intensity curves such as those shown in the preceding figure are depicted in Fig. 4a also but rotated to their respective zenith angles. While the curves assume an infinite material, the dashed line indicates the side view of a half-nanowall cutting across the high intensity regions of NIR intensity curves. Along the vertical, there is no conversion. This argument satisfies the spatial segregation of the UV and NIR components observed in the CL results. The same is illustrated with more clarity, color and a single nanocavity in Fig. 4b. Along the vertical axis the UV emanates out from the nanowalls unconverted as shown by the blue arrow. Near the horizontal axis, the converted NIR encounters the boundary of the nanowall and either refracts into the nanocavity between walls or directly emerge out. The escaped wave can strike the adjacent nanowall and either re-enter the material or get reflected. Near the vertical axis, some of the obliquely incident NIR onto the slanted interface will totally internally reflect and emanate along with the UV from the flat apex region of the nanowall. Notice that the emission point is placed near the bottom of the nanowall. This is considering the fact that at high electron beam energy, the penetration depth is also high. However, if we consider lower incident electron beam energies, the penetration depth is smaller and hence the emission point can be assumed to be located near the tip of the nanowall and hence it effectively has a smaller material region around. In this case, the UV will not have enough distance to travel for conversion into NIR and hence mainly UV will emanate from the walls. Thus, this construction and argument qualitatively explains, the spatial segregation of UV-NIR CL emission with changing electron beam energy discussed in the first section. From this analysis, it is evident that by constructing specific geometries of the material, it is possible to extract the NIR emission from specific regions on the surface before reconversion. The geometrical shape and physical dimensions determine which frequency eventually emerges from a particular region on the surface.

**Figure 5:**
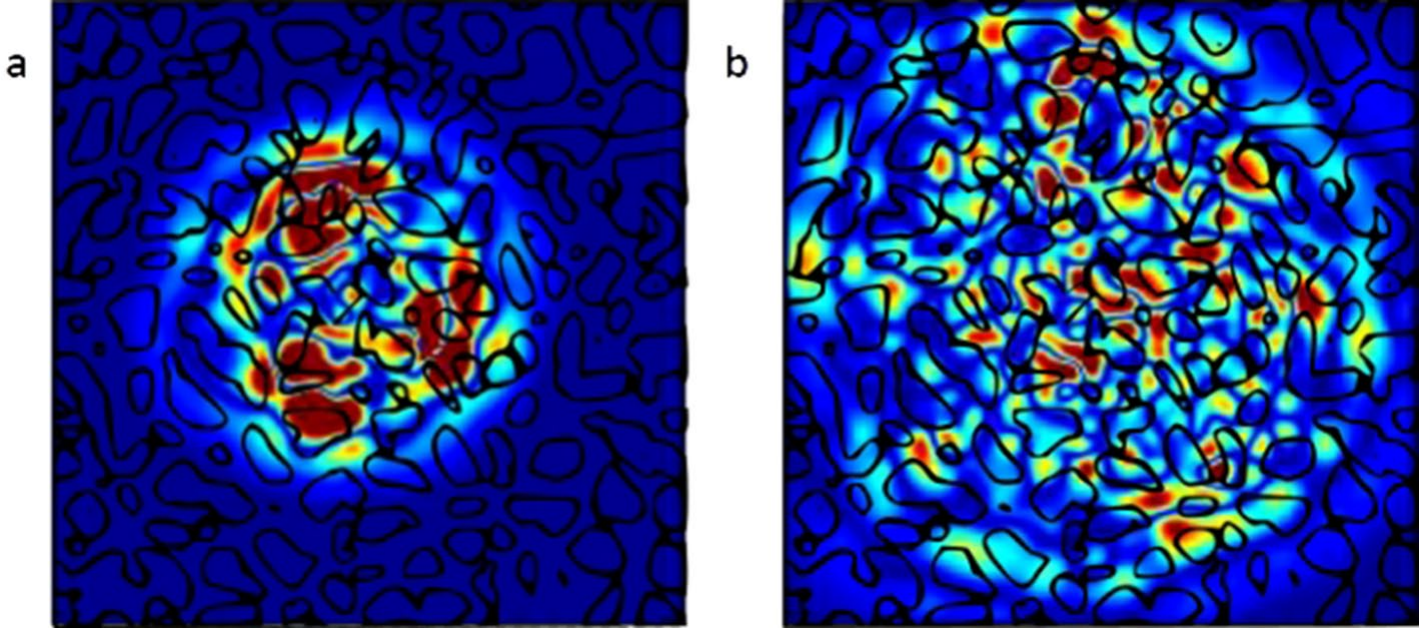
Pulse propagation in NwN morphology setting up whispering gallery type modes for a short duration (top view, snapshots at two instants)—single dipole source used. Time instant of (**a**) precedes that of (**b**). Red is highest field intensity and blue is lowest. See Supplementary Movie S1 for full animation of the simulation with modelled material morphology.

Next we consider the matter of lasing action apparent from the CL spectra result at cryogenic temperature. The fact that the sharp monochromatic peak observed at 692 nm emanating from the nanocavities did not show up in the results taken at room temperature is well noted and needs to be investigated further. Here, we look into the role of NwN morphology that can lead to the formation of laser-like cavity-resonators that may lead to the amplification of certain wavelengths (please note that here, the cavity-resonators are rather the nanowalls as the gain medium and not the previously talked nanocavities). A recent report^16^ has shown that light trapped in a micro or nanocavity sustains whispering gallery type resonance modes. It is important to note that in pyramidal structures or any appropriate 3d nanostructure, the lasing modes need not be confined only in one plane. 3D periodic orbits are also possible^17^ and also demonstrated for a variety of even deformed cavity shapes^18^. ZnO nanocavities which also have a wurtzite structure as that in GaN, have been used to demonstrate a UV nanolaser proposing helical whispering gallery type modes as the mechanism of lasing^19^. We performed a numerical simulation for a pulsed point dipole source acting as a source of superimposed waves with multiple frequencies considering the plan view of the NwN morphology extracted from the FESEM image. Two snapshots from the time evolution of the simulation results are shown in Fig. 5. The main implication of the result is that the modes persist for a while but due to irregular changes in the width and shape of polygonal walled network, the propagation is less persisting as compared to that of an unconnected waveguide cavity similar to a microdisk. Overall, our simulations suggest that the persistence of the modes is at-least expected from the NwN morphology for some duration if not for very long. Though the link of the amplification of a certain wavelength at cryogenic temperature remains unclear. A deeper analysis of the pulse propagation may reveal if modes of any particular frequency are preferred over others.

The unique experimental observations of frequency conversion and increase or decrease in the intensity of individual peaks or total emission as revealed by CL spectra are very interesting and the underlying mechanism can be attributed to a few possible processes, which may also be operating simultaneously such as optical modulation due to interference of fundamental beams from the sample, change in the optical susceptibility due polarization effects, variation in the non-linear properties due to stress induced by lattice mismatch at the GaN/Al_2_O_3_ interface, random lasing etc. At present, it appears that the Purcell effect, which enhances the spontaneous emission rates^20,21^ for emission from dielectric cavities at higher photon density of states (DOS), may be at work along with shortly persistent WGMs within a random lasing scenario while enhancing the nonlinear interactions^22^ in our system as well. Random lasing can operate either by resonant (field) feedback or non-resonant (intensity) feedback. Random lasers based on direct band-gap semiconductors have been demonstrated earlier. Several materials such as ZnO, GaAs, GaN, in the form of powder, film, nanowire, nanorod, nanoneedle have shown random lasing by distributed feedback. Laser peak emerging from a smooth gain profile is often an indication of resonant feedback^23^. A UV nanolaser was made using a single ZnO nanowire^24^. Among ordered and disordered array of ZnO nanorods it was found that the disordered configuration is better at lasing^25^. Further less threshold for lasing is observed, if the seminconductor is accompanied by nanoparticles such as TiO_2_^26^. GaN based random lasing has also been demonstrated in nanocolumn geometries^27^ and quasi-crystal nanorod arrays^28^.

## Conclusion

Cathodoluminescence mapping and spectral analysis of GaN NwN reveal spatially separated emission of UV and NIR frequency bands. Theoretical modelling and geometrical considerations for GaN nanowall network morphology reveal that UV frequencies undergo spontaneous parametric down conversion (SPDC) into NIR frequencies as a function of zenith angles and electron beam energy (penetration depth) resulting in the observed spatial separation of UV and NIR bands. At low temperature, an intense monochromatic peak emerges from the NIR band as the beam energy is increased. We believe that our results are of general nature, not specific to our material or system and may be observed in other nanostructured cavity semiconductors that have nonlinear polarization, where any two dominant emission bands can interact with each other to give similar results. Resolving the individual contributions from each of the above processes by experiments and simulations can enable a deeper understanding of the underlying mechanisms. Hence, we expect extensive follow-up research studies to confirm our predictions. Since controlling the nanostructure morphology with a variety of shapes and sizes can be achieved by kinetic or lithographic routes, this can be a major approach to obtain specific frequency range emissions from specific regions at nano or micro scales. We should also be able to tune the emission frequencies by making In_x_Ga_(1−x)_N alloys, yielding continuously varying band-edge emission which is evident from established use of GaN and its alloys in light-emitting diode applications^29–31^. Our apparatus is limited to detection of only CL intensity mapping, but we hope these results will generate the interest of research groups who can study polarization or directivity of the CL emission, that may throw up more exciting results. Based on our present results, we envision highly directional nano-torches/nano-beams/nano-lighthouses of light with tunable frequencies. This approach may be a precursor to obtaining highly intense laser sources of ultra-small dimensions packed at a high density of over 10^8^/cm^2^, where each source is addressable by appropriately deflecting the incident electron beam.

## Materials and methods

### Sample preparation and experimental techniques

Plasma Assisted Molecular beam epitaxy (SVT, USA) was used to grow high quality single crystal thin films of GaN on c-plane sapphire substrate. Prior to the growth, sapphire substrates were cleaned via chemical methods, degassed at 500 °C in the preparation chamber at 1 × 10^−9^ Torr vacuum for 60 min, and then again degassed in ultra-high vaccum (3 × 10^−11^ Torr) in the growth chamber for 30 min at 800 °C. Characteristic Kikuchi lines were observed in RHEED pattern to confirm an atomically clean substrate surface before commencement of growth. Substrate temperature of 630 °C was employed with N2 gas flow rate of 4.5 sccm during growth duration of 3 h with a RF forward power of 375 W, and the Ga Knudsen cell at 1000 °C. Morphological images were acquired using Field Emission Scanning Electron Microscope (Quanta 3D FEG, FEI, Netherlands) at different magnifications and at different operating voltages. Gatan monoCL4 system with a beam spot of ≈ 1 nm was used to detect spatially-resolved CL emission maps and spectra in the range of 250–900 nm (PMT Detector as well as CCD for parallel spectroscopy). This CL detection system consists of a diamond turned collection mirror and is equipped with digital beam control and image processing features provided by DigiScan II System. The inbuilt auto-calibrating spectrometer is equipped with high efficiency achromatic optics. It has a single grating with choice of blaze and dispersion. It has direct optical coupling to the chamber mounted monochromator. Sufficient care has been taken to ensure that the spectral and spatial emission observations are not due to measurement artifacts.

### Modelling and numerical techniques

The non-linearly coupled equations for three-wave mixing were solved in MATLAB using ODE solver. The pulse propagation simulation for emission from NwN morphology was performed in MATLAB by using 2D-FDTD method. Absorbing boundaries were implemented via ‘perfectly matched layers’ on all four sides. A Gaussian pulse was used to simulate a broad-band short pulse in time. Courant’s stability condition was used to choose the timesteps. Sufficient care was taken to set the least feature size (2–4 nm) ensuring that the shortest wavelengths present in the simulation are well resolved. The largest spatial extent varied from 900 to 1800 nm accordingly. The simulations were made to run for about thirty femtoseconds.

## Supplementary information

Supplementary Video.

Supplementary Information.
