# Supplementary material for: Observation of monochromatic and coherent luminescence from nanocavities of GaN nanowall network
Source: Sci Rep. 2021 Apr 30;11:9368. doi: 10.1038/s41598-021-88660-3 (PMC8087758; doi:10.1038/s41598-021-88660-3)
Supplement: Supplementary file 2 — Supplementary Information. [file 41598_2021_88660_MOESM2_ESM.docx]

**Supplementary Information for**

Observation of monochromatic and coherent luminescence from Nanocavities of GaN Nanowall Network

Danish Shamoon, Kishor Upadhyaya, Sonnada M. Shivaprasad

Correspondence to: Sonnada M. Shivaprasad

Email: smsprasad@jncasr.ac.in

**This PDF file includes:**

Supplementary information text

Supplementary Fig. S1 to S4

SI References

(Supplementary Movie S1 is available online)

Supplementary Information Text

**Nonlinear interaction of UV and NIR emission.** GaN has wurtzite crystal structure. The wurtzite structure is non-centrosymmetric i.e. it lacks an inversion center. As a consequence of this, there is a net charge separation in the unit cell which essentially gives it a permanent dipole moment per unit volume or commonly known as spontaneous polarization. A non-zero second order susceptibility (χ^(2)^) is also an associated consequence. Material polarization as a response to an electric field is mathematically written as linear and non-linear contributions as follows:

$$P_{L}=\varepsilon_{o}\chi^{(1)}E$$

$$P_{NL}=\varepsilon_{o}{(\chi}^{(2)}E^{2}+\chi^{(3)}E^{3}+\ldots)$$

$${P=P}_{L}+P_{NL}$$

The electrical displacement can be written as $D=\varepsilon E+P_{NL}$, where $\varepsilon=\varepsilon_{o}\varepsilon_{r}$ and $\varepsilon_{r}=1+\chi^{(1)}$. So, the wave equation obtained from Maxwell’s equations can be written as

$$\nabla^{2}E-\nabla\left( \nabla.E \right)-\mu\varepsilon\frac{\partial^{2}E}{{\partial t}^{2}}=\mu\frac{\partial^{2}P_{NL}}{{\partial t}^{2}}$$

Higher than second order terms are ignored. This wave equation is thereby employed to construct the arguments for spatio-spectral behavior of the CL emission from w-GaN theoretically.

Based on previous publications [14,15], we note that N^th^ order nonlinear process involves interaction among N+1 frequencies. Hence, a χ^(2)^ process involves interaction among three frequencies, also known as three-wave mixing. It can generate new frequencies at twice of any frequency, sum and difference of any frequency and optical rectification or amplification which can also be called zero frequency. Not all of these responses are generated in the material efficiently due to phase matching constraints and energy conservation. The resultant frequencies can further interact to produce more new frequencies but newer amplitudes will be even smaller than that of their generating frequencies. The second order susceptibility χ^(2)^ is a 3 x 3 x 3 tensor which is reduced to a 3 x 6 matrix using Kleinman symmetry operations. The reduced form is known as the contracted notation *d*_iM_. Its elements further depend on the crystal symmetry to be non-zero. For hexagonal symmetry only three of its elements are non-zero out of which only two make it to the final form of coupled-differential equations (shown below) derived from the general wave equation including the nonlinear polarization by using the condition ω_3_ = ω_1_ + ω_2_ for energy conservation and k_3_ = k_1_ + k_2_ for phase matching, where ω_3_ can be considered as the peak frequency of UV band and ω_1_ and ω_2_ as the peak frequency of NIR band. It must be noted that w-GaN has birefringence (refractive index is different for specific polarization of light and also its direction within the crystal) that can fulfill this phase matching condition. Extended Data Figure 3 shows a visual depiction of the energy conservation condition ω_3_ = ω_1_ + ω_2_ which is equal to 1/λ_3_=1/λ_1_ + 1/λ_2_.

The coupled equations are

$$\frac{\partial E(\omega_{1})}{\partial s}=m*E\left( \omega_{3} \right)E^{*}(\omega_{2})$$

$$\frac{\partial E(\omega_{2})}{\partial s}=m*E(\omega_{3})E^{*}(\omega_{1})$$

$$\frac{\partial E(\omega_{3})}{\partial s}=m*2E(\omega_{1})E(\omega_{2})$$

where *m* is $d_{31}sin\theta$ for type 1 process and $d_{15}\{{(cos}^{2}\theta*cos\varphi*cos2\varphi)+(sin\theta*cos\theta*sin\varphi*cos\varphi)\}$ for type 2 process, *θ* and *ϕ* are azimuthal and polar angles respectively. Type 1 and type 2 processes are most common in negative uniaxial materials such as w-GaN. However, more such processes can be possible. They differ in whether the directions of the pump, signal and idler waves involved in nonlinear interaction are ordinary or extra-ordinary. The angular part *m* for both types are plotted as a 3D surface in Supplementary Fig. S4 that illustrates the strength of nonlinear interaction along any given direction in the crystal.

When we choose ω_3_ to be the higher frequency and the other two as the lower frequencies then this nonlinear interaction specifically refers to spontaneous parametric downconversion (SPDC) in which a high frequency wave is converted to two low frequency waves. The opposite is called upconversion. Both of these occur along the path of the wave in the crystal so that the intensity of each wave varies with distance as well as direction from the point of origin of the high frequency wave. It is worth mentioning that our calculation using the classical model requires trace amounts of NIR frequencies for the initial condition. For example, we used initial amplitudes (0.01, 0.01, 1) for (ω_1_, ω_2_, ω_3_) to generate the intensity vs radial distance graph in Extended Data Figure 4 but if we use (0, 0, 1) then NIR is not generated. However, if a quantum mechanical approach is used then NIR emission can be generated directly from the UV by spontaneous parametric down conversion [32] (SPDC). Nevertheless, trace amounts of frequencies in the entire detection range will be present due to the transition radiation emitted, as the electron beam enters the material [33], from which NIR frequencies can be picked up by the strong UV emission for nonlinear interaction.


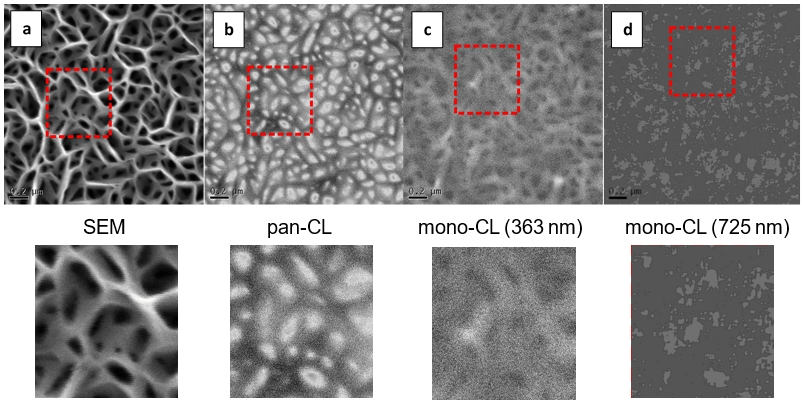


Fig. S1. FESEM-CL maps of w-GaN NwN network at room temperature and applied voltage 25 kV. (A) SEM image, (B) panchromatic CL image (250-900 nm), (C) monochromatic CL image (363 nm), (D) edited monochromatic CL image (725 nm). Boxed regions (red) in CL images represent the same region also shown separately below each of the images (they are slightly shifted upwards in C and D because the electron beam moved to a slightly different region while capturing those regions), Width of the box is 600 nm, Scalebar in bottom left corner of images measures 200 nm.


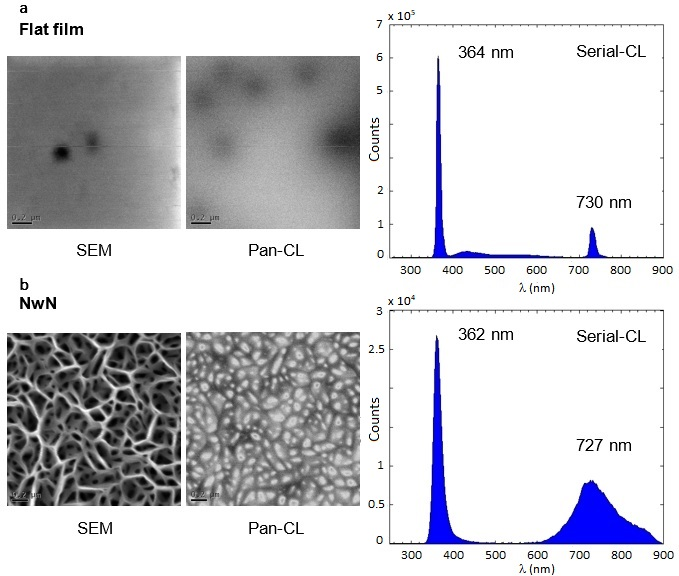


Fig. S2. Comparison of CL from w-GaN flat-film and NwN. (A) CL spectra and panchromatic-CL map with corresponding SEM image at room temperature for w-GaN flat film with 30 kV applied voltage, (B) NwN with 25 kV applied voltage, Scalebar in bottom left corner of the images is 200 nm.


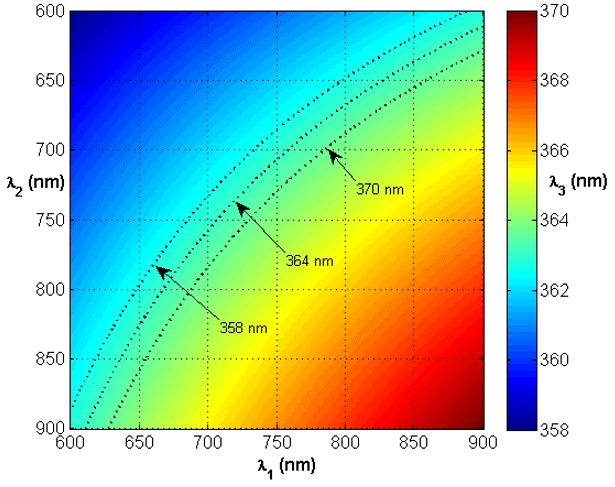


Fig. S3. Energy conservation ω_3_=ω_1_+ω_2_ roughly allows lower frequencies to fall within 600 nm to 900 nm wavelength range.


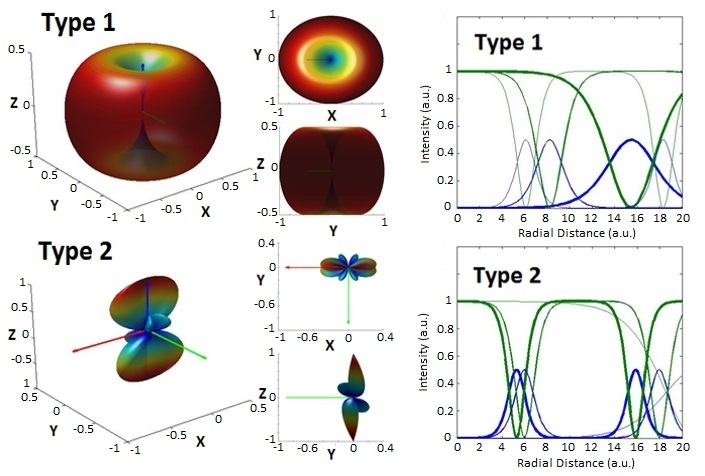


Fig. S4. Angular part of strength of frequency conversion from different viewing angles (red to blue is high to low) shown on left side and on the right is three wave mixing showing graphs of Intensity vs distance travelled inside material for different zenithal angles θ which are (20, 40, 60) degrees. Initial Amplitudes for (ω_1_, ω_2_, ω_3_) in both cases are [0.01 0.01 1]. Blue is for ω_1_ and ω_2_ (they overlap) and Green is for ω_3_. Thicker line is for smaller angles and thinner line is for larger angles.

**SI References (also included in main references)**

14. Boyd, R. W. *Nonlinear optics*. (Academic press, 2019).

15. Catalano, J. Spontaneous Parametric Down-Conversion and Quantum Entanglement. (2014). [pdxscholar.library.pdx.edu]

32. Podoshvedov, S. A., Noh, J. & Kim, K. A full quantum theory of parametric down conversion and its application to coincidence measurements. *Journal of the Korean Physical Society* **47,** 213–222 (2005).

33. Coenen, T., Brenny, B. J., Vesseur, E. J. & Polman, A. Cathodoluminescence microscopy: Optical imaging and spectroscopy with deep-subwavelength resolution. *Mrs Bulletin* **40,** 359–365 (2015).
